# Preparative Purification of Linarin Extracts from *Dendranthema indicum* Flowers and Evaluation of Its Antihypertensive Effect

**DOI:** 10.1155/2014/394276

**Published:** 2014-11-23

**Authors:** Yin Qiaoshan, Chen Suhong, Su Minxia, Mi Wenjia, Li Bo, Lv Guiyuan

**Affiliations:** ^1^Zhejiang Chinese Medical University, Hangzhou, Zhejiang 310053, China; ^2^Wenzhou Medical University, Wenzhou, Zhejiang 325035, China; ^3^College of Pharmaceutical Science, Zhejiang Chinese Medical University, Binwen Road No. 548, Binjiang District, Hangzhou, Zhejiang 325035, China

## Abstract

*Background*. Preliminary research showed that linarin (LIN) might have a relationship with the antihypertensive effect of *Dendranthema indicum* flowers. However, the preparative method for LIN enriched extract from *Dendranthema indicum* flowers was not clear and its antihypertensive effect was not confirmed. In this study, we designed a series of experiments to develop an efficient method for purification of LIN extracts and confirm the possibility of LIN extracts to be an antihypertensive drug. *Materials and Methods*. HPLC-VWD/DAD were used in the process of developing purification method. The antihypertensive effect of LIN extracts was tested by CODA Mouse & Rat Tail-Cuff Blood Pressure System; western blot and biochemical analysis were used to investigate mechanism and toxicity. *Results*. The content and recovery of LIN reached 55.68 ± 2.08% and 66.65 ± 1.73%, respectively, through solid-liquid extraction. The composition of product was stable through the analysis of fingerprint. Chronic administration of LIN extracts reduced blood pressure obviously which had a relationship with the inhibition of renin-angiotensin system (RAS) in kidney and the function indexes of kidney and liver had no variations. *Conclusions*. The preparation method was simple, low-cost, and stable, and it was fit for industrial application. The LIN prepared by this method had the potential to be an antihypertensive drug.

## 1. Introduction

*Dendranthema indicum* flowers have been used for hundreds of years in Chinese traditional medicine. It had been proved to be effective for hypertension treatment [1]. As a natural plant, it is an ideal source to develop effective drug as antihypertensive because of its little additional effects.

The flavonoids in it were thought to be a key role in the effect as antihypertensive [2, 3]. LIN is rich and important in the identified flavonoids from* Dendranthema indicum* flowers. The activities of LIN which have been proved were diverse, containing anti-inflammatory, antitumor, antioxidant, antibacterial, sedation, analgesia, and antihypertension [4–14]. However, it was still unclear whether LIN is one of the main substances for antihypertensive in* Dendranthema indicum* flowers until our laboratory research group implied that the LIN enriched extracts combined with luteolin could lower blood pressure in spontaneously hypertensive rats (SHRs) [11].

Nevertheless, LIN used in the study was purchased from a company, so the purification method for LIN was not clear [11]. And it would be an obstruction to develop LIN to be an antihypertensive drug. According to prior papers, the separation methods for LIN from* Cirsium setosum* [15], leaves of* Turpinia arguta* [16], and* Dendranthema indicum* [17] have been published, but the separation methods were all not proper for large-scale preparation. So it is necessary to develop a high-efficient, low-cost, and environment-friendly method for large-scale preparation of LIN from* Dendranthema indicum* flowers.

It was reported that the LIN (50%) from Flos Chrysanthemi flowers at 25 mg/kg was not effective as antihypertensive [11]. But it was uncertain whether it was effective with high dosage of LIN. So it is meaningful to ensure the effect of LIN as antihypertensive at large dose.

The purpose of this study was to develop a method which could afford approach for industrial application for enrichment of LIN by solid-liquid extraction. Quantitative analyses of LIN extracts were taken to evaluate the extraction characteristic of different polar solvents. The antihypertension effect of LIN extracts on SHR was measured to assess the property of lowering blood pressure. The antihypertensive mechanism was determined by western blot. And the effects of LIN extracts on the function of kidney and liver were also tested.

## 2. Experiment

### 2.1. Chemicals, Reagents, and Samples

Linarin standard was purchased from National Institutes for Food and Drug Control. The purity of LIN was higher than 98%. Ethanol (analytical grade), petroleum ether (analytical grade), and ethyl acetate (analytical grade) were purchased from Shanghai Huadong Reagent Co., Ltd. Deionized water was purified by a DW100 purification system from Hangzhou Yongjieda Pure Technology Co., Ltd. SHRs were purchased from Beijing Weitonglihua Experimental Animal Technical Co., Ltd. The* Chrysanthemum indicum* flowers were purchased from Zhejiang Chinese Medical University, Traditional Chinese Medicine Decoction Pieces Co., Ltd. The antibodies in this paper were purchased from Santa Cruz Biotechnology, Inc.

### 2.2. Preparation of the Crude Extracts of* Chrysanthemum indicum*

2.5 kg dried* Chrysanthemum indicum* flowers were extracted with 25 L 75% ethanol-aqueous solution at 100°C for 1.0 h in a traditional Chinese medicine extracting machine (YF-40, Beijing Donghuayuan Medical instrument Co., Ltd.), repeated three times. The extracted liquids were pooled and concentrated under vacuum to 2.5 L. Then the precipitate which was obtained by centrifuging at 3500 rpm for 5 min was collected. Through the detection, it was found that the LIN in the precipitate reached above 95% of the total LIN. So the precipitate was used as raw material (RM) for the purification of LIN. The weight of dried RM was 98 g.

### 2.3. HPLC Analysis of LIN

The Agilent liquid chromatographic system (1200 series) comprising quat pump, a VWD detector, and LC solution software was employed to do quantitative analysis of LIN. The XB-C18 column (4.6 × 250 mm, Welch Materials, Inc.) was used at a column temperature of 25°C. The mobile phase was methanol-water-acetic acid (50 : 49.95 : 0.05, v/v/v). The VWD was set at 334 nm and flow rate was set at 1.00 mL/min. The chromatographic peak of LIN was identified by comparing its retention time and UV spectra with LIN standard. All solutions were filtered through a 0.45 *μ*m membrane filter prior to use. The calibration curve was established with a good linear relationship over the range of 20–500 *μ*g/mL. The regression curves for LIN were *y* = 9.144*x* − 3.006, where *y* is the peak area of LIN and *x* is the concentration of LIN (*μ*g/mL).

### 2.4. The Solubility of LIN in Different Polar Solvents

It was necessary to know the solubility of LIN in different polar solvents before solid-liquid extraction. The experiments were carried out as follows: excessive RM were added into a sealing conical flask with 50 mL different polar solvents; then the solution was stirred sufficiently with a magnetic stirring apparatus and then centrifuged at 3500 rpm for 5 min. The supernatant was collected to evaluate the amount of impurities by observing the color of the solution with eyes and determine the amount of LIN by HPLC which dissolved in solvents.

### 2.5. The Purification of LIN by Solid-Liquid Extraction

According to the results of the experiments described in Section 2.4, four different polar solvents were selected for solid-liquid extraction. They were petroleum ether, ethyl acetate, ethanol, and ethanol-water solution (40%, v/v). In order to study the effect of solid-liquid extraction with these solvents on the purity of LIN, the experiments were carried out as follows: 40 g RM was mixed with 50 mL solvents; then the solution was stirred sufficiently with a magnetic stirring apparatus and then centrifuged at 3500 rpm for 5 min to collect the precipitate. This extraction process was repeated two times with petroleum ether, ethyl acetate, ethanol, and ethanol-water solution (40%, v/v), respectively. Very little part of the precipitate after each extraction was collected to determine the purity of LIN. According to the results, the simplest process was chosen to separate the LIN.

### 2.6. The Effect of Temperature on the Stability of Method

Generally, the temperature is an important factor to affect the solubility. In order to study the influence of temperature on the product, the experiments were carried out as follows: the process of solid-liquid extraction was taken at 4°C and 30°C separately. In the experiment at 4°C, all solvents were precooled in refrigerator. The extraction process was carried out in a cold bath which was refrigerated by ice with the help of thermometer, and the centrifugation was taken in a refrigerated centrifuge (Heraeus Biofuge Stratos, Germany). In the experiment at 30°C, all solvents were put in a room at 30°C at the control of air-condition in advance. Then all of the process of extraction was completed in the same room.

### 2.7. The Assays of Scale-Up

15 kg* Chrysanthemum indicum* flowers were extracted to get RM. Then the RM was purified under the optimal conditions. The content of LIN in the product was determined by HPLC. The fingerprint of LIN was examined by HPLC-DAD; the method was similar as described in Section 2.3 except for the mobile phase and wavelength. The mobile phase was methanol (B)/acetic acid solution (0.2%/D) in a linear gradient as follows: 0–10 min (B40%–50%); 10–35 min (B50%–100%); 35–50 min (B100%). Wavelength is 200–360 nm.

### 2.8. The Antihypertension Effect of LIN in SHRs

Ten Wky rats which are 12 weeks old and forty male SHRs which are 12 weeks old were divided into five groups. The Wky group (group 1, G1) and vehicle group (group 2, G2) were given distilled water orally. The positive control group (group 3, G3) was given valsartan (8 mg/kg) orally. The LIN groups were given LIN 75 mg/kg (group 4, G4) and 150 mg/kg (group 5, G5) orally, respectively. The CODA Mouse & Rat Tail-Cuff Blood Pressure System (KENT Scientific Co., Connecticut, USA) was used to determine the systolic, diastolic, and mean arterial blood pressures (SBP, DBP, and MAP for short) in conscious rat to evaluate the antihypertension effect of LIN. At 2 hours after administration, the blood pressure of each rat was tested for three consecutive times on the purpose of getting its mean value.

### 2.9. Western Blot

In accordance with prior papers, the kidney is an important organ for the initialing and progression of hypertensive, so it is necessary to observe the drug target in kidney. The kidney of SHRs obtained after six-week continuous treatment was ground in liquid nitrogen to extract protein. Then the change of the protein determined by western blot was used to insure the potential drug target. The western blot was similar as described by Maffei et al. [18].

### 2.10. Determination of Functional Indexes of Kidney and Liver

After six-week continuous treatment, whole blood was collected from the orbit. Then it was centrifuged to collect serum. Alanine transaminase (ALT), aspartate transaminase (AST), blood urea nitrogen (BUN), and creatinine (CRE) in serum were detected with kits (Ningbo Medical System Biotechnology Co., Ltd.) in a biochemical autoanalyzer (TBA-40FR, Toshiba).

### 2.11. Statistical Analysis

All values were expressed as mean ± standard deviation and subjected to one-way analysis of variance (ANOVA). Difference was considered to be statistically (*P* < 0.05). SPSS 17.0 for windows was used for analysis.

## 3. Results and Discussion

### 3.1. The Solubility of LIN in Different Polar Solvents

**Figure 1:**
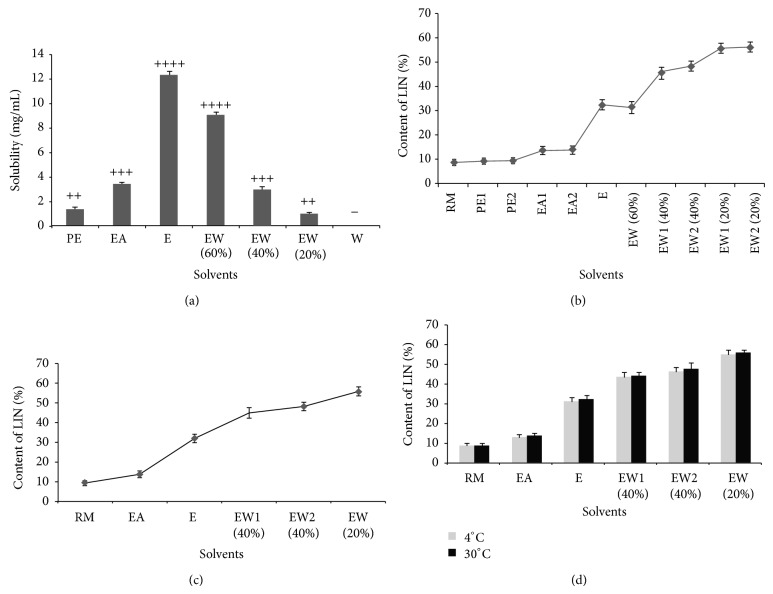
The purification of LIN from* Dendranthema indicum *flowers. The data were expressed as mean ± error (*n* = 3). (a) The solubility of LUT and impurity in different polar solvents. “+” and “−” stand for different shade colors to evaluate the solubility of impurity. (b) The preliminary research about the influence of different solvents on the purification of LIN. (c) The simplification of the purification of LIN with solvents. (d) The influence of temperature on the purity of LIN separated by the solid-liquid extraction. (RM: Raw Material, PE: Petroleum Ether, EA: Ethyl Acetate, E: Ethanol, EW: Ethanol-Water, W: Water).

The tested extraction solvents were chosen on the principles of low toxicity and proper polarity distribution. Low toxicity could supply a safe and friendly environment in the process of production. Proper polarity distribution meant these solvents should have representative in polarity from low to high, which could be used to study the solubility of LIN in different polar solvents better. In consideration of these two factors, petroleum ether, ethyl acetate, ethanol, ethanol-water (60%), ethanol-water (40%), ethanol-water (20%), and water were chosen. The results were shown in Figure 1(a). It showed that the capacity of these solvents to dissolve the LIN was not very strong; the relative stronger two were ethanol and 60% ethanol-water solution. The ability of these solvents to dissolve impurities was evaluated by observing the change of solution color. The results demonstrated that water which was almost colorless had very weak dissolving ability for impurities. After making comprehensive consideration of the results of dissolving ability of linarin and impurities, the petroleum ether, ethyl acetate, ethanol, ethanol-water (60%), ethanol-water (40%), and ethanol-water (20%) were selected to do further study as extraction solvents, but the use of ethanol and 60% ethanol-water solution should be as little as possible.

### 3.2. The Effect on the Purity of LIN by Solid-Liquid Extraction with Different Polar Solvents

The results of the effect on the purity of LIN by solid-liquid extraction with different polar solvents were shown in Figure 1(b). It showed that the first time extraction with ethyl acetate, ethanol, 40% ethanol-aqueous, and 20% ethanol-aqueous solution increased the purity of LIN obviously while with petroleum ether it just increased a little. However, the purity of LIN in the second time extraction with ethyl acetate had no obvious change while with 40% ethanol-aqueous solution it still enhanced a little. On the other hand, 60% ethanol-aqueous solution reduced the purity of LIN in the first time extraction. According to these results, the process of extraction was reduced to one time with ethyl acetate, one time with ethanol, two times with 40% ethanol-aqueous solution, and one time with 20% ethanol-aqueous solution, respectively. The verification results of this reduced process were shown in Figure 1(c). It indicated that the reduced process of extraction was completely feasible and the purity of LIN reached 55.68 ± 2.08% and the recovery of LIN reached 66.65 ± 1.73%.

### 3.3. The Influence of Temperature on the Stability of Product

The method obtained in Section 2.5 was established on the basis of the solubility of substances in different solvents, so the environment temperature was an important factor for the stability of the method. In this study, 4°C and 30°C were selected to represent the lowest temperature and highest temperature, respectively, in a normal indoor environment to study the stability of method. The results were shown in Figure 1(d). It showed that the change of purity was not obvious after each extraction, and the final purity of LIN reached 55.28% and 56.24% at 4°C and 30°C, respectively. It indicated that the temperature range in indoor environment did not affect the quantity of product obviously.

### 3.4. Large-Scale Preparation of LIN

**Figure 2:**
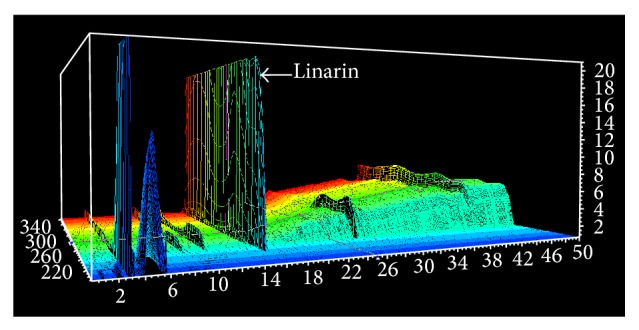
The fingerprint of product by HPLC-DAD.

The large-scale preparation of linarin was carried out under the optimal condition. At last, the content of LIN in products reached 55.33 ± 0.85% and the recovery of LIN reached 64.8 ± 1.2%. The fingerprints of LIN in three repeated experiments were shown in Figure 2. The analysis of fingerprints demonstrated that the main component in the products had no obvious change (date not shown). It indicated that the method was feasible enough for the purification of LIN.

### 3.5. The Antihypertension Effect of LIN in SHRs

**Figure 3:**
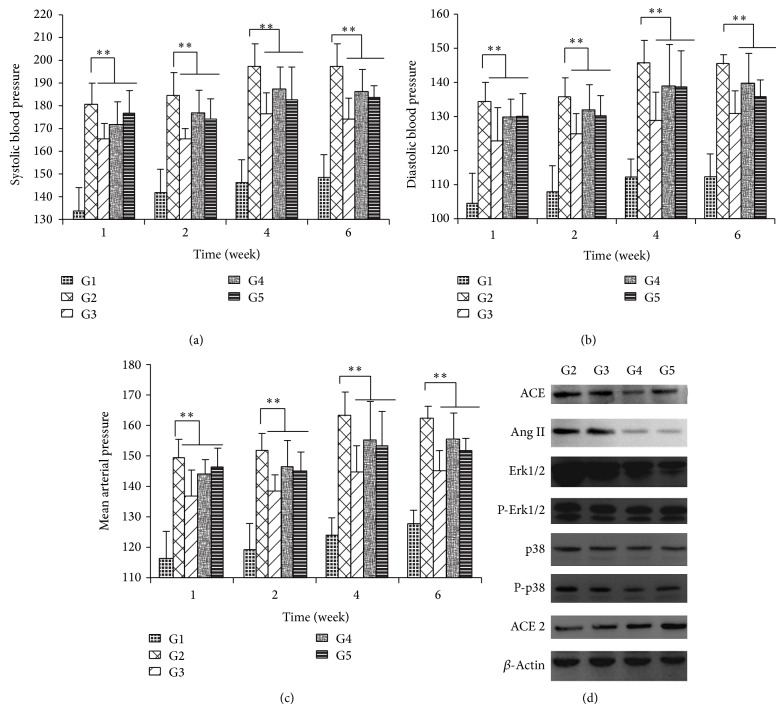
The antihypertensive effect and mechanism of LIN extracts in SHRs. (a) The effect on SBP. The data were expressed as mean ± error (*n* = 10). (b) The effect on DBP. The data were expressed as mean ± error (*n* = 10). (c) The effect on MAP. The data were expressed as mean ± error (*n* = 10). (d) The effect of LIN extracts on signaling pathway in kidney (^**^ *P* < 0.01, G1 = Wky group, G2 = vehicle group, G3 = Valsartan group, G4 = LIN 75 mg/kg group, and G5 = LIN 150 mg/kg group).

We had reported [11] that 25 mg/kg LIN did not lower the SBP or DBP of the SHR rats significantly compared to vehicle-treatment group. In this experiment, the dosage was enlarged to 75 mg/kg and 150 mg/kg. As shown in Figure 3, both 75 mg/kg and 150 mg/kg LIN decreased the SBP obviously (*P* < 0.01, Figure 3(a)), and the average depressurization rate of SBP in six weeks reached 6.66% and 5.77%, respectively. In addition, both DBP and MAP of SHRs also were decreased obviously by 75 mg/kg and 150 mg/kg LIN (*P* < 0.01, Figures 3(b) and 3(c)), respectively. However, the average depressurization rate by 150 mg/kg was smaller than by 75 mg/kg. It indicated that LIN had antihypertension properties and might have the best effect with a certain dosage between 25 mg/kg and 150 mg/kg by combination of the results reported in the last paper and in this paper.

### 3.6. The Potential Drug Targets for Antihypertension by LIN in SHRs

The intrarenal RAS plays a key role in the development of blood pressure in SHR [19]. Angiotensin-converting enzyme 2 (ACE2) has been proved to be a novel enzyme involved in the regulation of the RAS [20]. In this study, the influence of LIN on the RAS was determined. As shown in Figure 3(d), the ACE and Ang II in kidney were reduced obviously in the treatment of LIN. According to prior papers, Ang II could improve the expression of ACE and inhibit the expression of ACE2 by activating the phosphorylation of p38 MAPK and Erk 1/2 [21]. Under the interference of LIN, the feedback regulation of Ang II for ACE and ACE2 was suppressed by inhibiting the signaling pathway of p38 MAPK and Erk 1/2. In conclusion, the antihypertensive effect of LIN, at least in part, was produced by inhibiting ACE and Ang II and promoting ACE2 in kidney of SHR.

### 3.7. The Influence of LIN Extracts on the Functions of Kidney and Liver

**Figure 4:**
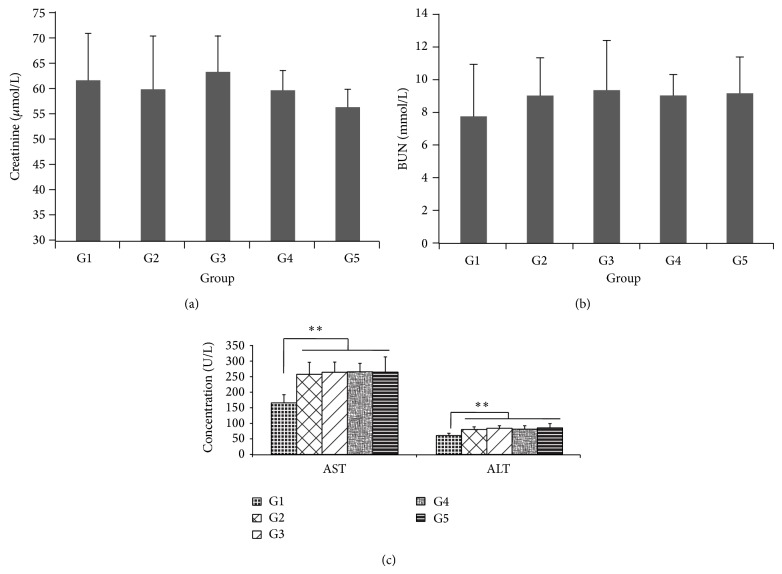
The biochemical analysis in serum. The data were expressed as mean ± error (*n* = 10). (a) The variation of CRE in serum. (b) The variation of BUN in serum. (c) The variation of AST and ALT in serum (^**^ *P* < 0.01, G1 = Wky group, G2 = vehicle group, G3 = Valsartan group, G4 = LIN 75 mg/kg group, and G5 = LIN 150 mg/kg group).

The effective dosage of LIN extracts was large in this study, so it was necessary to determine the influence of LIN extracts on the functions of kidney and liver. As shown in Figures 4(a) and 4(b), BUN and CRE in serum had no obvious variation compared with vehicle-treatment group; it indicated that LIN extracts in these dosages had no damage to kidney function. The determination of ALT and AST in serum (Figure 4(c)) also showed that the dosages of LIN extracts were safe for liver function. However, this experiment was not enough to judge the toxicity of LIN extracts; it just illustrated that LIN extracts at the dosages of 75 mg/kg and 150 mg/kg were safe for kidney and liver in a short time.

## 4. Conclusions

In this study, the method for the enrichment of LIN extracts from* Chrysanthemum indicum* flowers was established. Through detecting the solubility of LIN and impurities in different polar solvents, four solvents (ethyl acetate, ethanol, 40% ethanol-aqueous solution, and 20% ethanol-aqueous solution) were selected as extraction solvents. These four solvents which had low toxicity could be recycled easily to lower the cost. Further extraction experiments with these four solvents were conducted to gain the optimal parameters for the purification of LIN. It was proved that the quantity of product obtained at different environment temperature could be assured. Through the analysis of the product obtained in the scale-up experiments, we found that the purity and recovery of LIN and the other components of the product were stable. It indicated that the method for the enrichment of LIN was worthy of being studied for industrial application. The date in this study showed that the LIN extracts could lower blood pressure of SHRs. The results of western blot showed that LIN extracts could affect the expression of ACE/ACE2 in kidney. It implied that the antihypertensive mechanism of LIN extracts had a relationship with attenuating RAS in kidney. The determination of kidney and liver function showed that LIN extracts had no obvious toxicity in this six-week continuous administration for SHR. In short, LIN separated by the method developed in this paper had the potential to be an antihypertensive drug.
